# The healing power of music: a promising new avenue for cardiovascular health

**DOI:** 10.3389/fcvm.2023.1277055

**Published:** 2023-09-12

**Authors:** Solomon Bendayan

**Affiliations:** Internal Medicine, Jewish General Hospital, McGill University, Montreal, QC, Canada

**Keywords:** music, heart failiure, cardiac rehabiliation, pain managemant, music therapy

## Introduction

Cardiovascular diseases are commonly treated using non-pharmacological interventions, which are often the preferred first-line approach. Unfortunately, there appears to be a lack of utilization and understanding regarding the most effective non-pharmacological options available. The reasons for this knowledge gap are not entirely clear; however, the pharmaceutical industry's influence could be a contributing factor. Additionally, many non-pharmacological interventions for cardiovascular health may not be well-received by patients due to the burden they impose. For example, interventions such as smoking cessation, dietary changes, and exercise can be labor-intensive and unpleasant. A promising new non-pharmacological intervention for cardiovascular disease is the use of music.

Music has long been a source of comfort, inspiration, and entertainment for people around the world. But recent research suggests that it may have even more profound benefits, particularly when it comes to cardiovascular health. Music has been shown to impact the cardiovascular system through various mechanisms. One proposed mechanism is the effect of music on the autonomic nervous system (ANS). Studies have shown that music can modulate ANS activity, resulting in decreased heart rate and blood pressure (1). This effect may be mediated by the release of endogenous opioids, which have been shown to be involved in the modulation of ANS activity (2). Classical music with a slow beat has been shown to reduce heart rate and blood pressure, while classical music with a fast beat has been found to increase heart rate and blood pressure (3). These effects can be attributed to the ability of music to affect the release of hormones such as adrenaline and cortisol, which are associated with the sympathetic nervous system response, or oxytocin, which is associated with the parasympathetic nervous system response (4). In addition to its effects on the ANS, music has also been shown to impact the endothelial function of blood vessels. The endothelium is a layer of cells that lines the inside of blood vessels and is involved in the regulation of blood flow and vascular tone. Dysfunction of the endothelium is a hallmark of cardiovascular disease and is associated with increased risk of cardiovascular events. Studies have shown that music can improve endothelial function, possibly through the release of nitric oxide, a key regulator of vascular tone (5).

One of the most widely recognized clinical uses of music in the context of cardiovascular disease is the utilization of music therapy as part of cardiac rehabilitation. Music therapy has demonstrated efficacy in enhancing rehabilitation tolerance, elevating mood, and promoting adherence to rehabilitation exercises (6). Typically, the application of music therapy is directed towards either reducing anxiety and stress or fostering a motivational environment for rehabilitation. Randomized clinical trials have demonstrated a significant improvement in various health-related outcomes for patients that undergo cardiac rehabilitation with music therapy compared to patients who undergo cardiac rehabilitation without music therapy (7). Additionally, music therapy in post-cardiac surgery patients may decrease pain and anxiety (8). Finally, using music to diminish pain reduces the use of analgesic medication and reduces adverse effects of opioid analgesics such as falls and delirium.

In recent years, the use of music in invasive cardiac procedures has gained prominence as an effective non-pharmacological intervention. Hospitals can be a frightening place for patients, and the thought of undergoing a cardiac procedure can be incredibly anxiety-inducing. It's no surprise that patients often feel overwhelmed and stressed when faced with this daunting experience. Researchers have demonstrated significant reductions in anxiety and pain levels among adult patients who received music interventions before, during, and after surgery (9). Additionally, various types of music have resulted in improved patient satisfaction during surgical procedures (10). The effects of music listening on patients during cardiac catheterization were investigated and music was found to have a significant relaxing and calming effect for the patients (11).

The ensuing section pertains to the exploration of music's role within the domain of heart failure management. Heart failure management requires a multidisciplinary team and clinical trials typically focus on improving survival and decreasing exacerbations. However, heart failure is associated with many comorbidities such as depression, anxiety, and poor sleep quality. Despite the recognized negative impact of these comorbidities on patients' overall well-being and treatment outcomes, they are often overlooked or under-addressed in clinical practice. From a quality-of-life point of view, listening to music has been shown to improve various aspects such as anxiety, depression and sleep quality (12). Despite the growing evidence for the benefit of music therapy in heart failure, there are still some challenges to its implementation in clinical practice. One major issue is the lack of standardized protocols for music therapy, making it difficult to compare studies and establish best practices. Additionally, there is a need for more research on the optimal timing, frequency, and duration of music therapy interventions in heart failure.

The healing power of music has emerged as a promising new avenue for improving cardiovascular health. With numerous studies pointing towards its efficacy, music has demonstrated its ability to reduce stress, anxiety and even lower blood pressure and heart rate. Furthermore, music may contribute to the improvement of endothelial function, a critical element in upholding cardiovascular health. The potential of music therapy as a complementary intervention in managing cardiovascular diseases is indeed impressive and calls for more in-depth exploration. By incorporating music as a non-pharmacological intervention in cardiovascular disease management, healthcare providers can potentially improve outcomes and enhance the quality of life for their patients. The incorporation of music into the domain of cardiovascular health has unveiled a realm of novel opportunities, holding the promise of remarkable advancements in the foreseeable future.
